# Influences of Structural Empowerment and Demographic Factors on Nurses' Psychological Empowerment

**DOI:** 10.1155/2023/8827968

**Published:** 2023-10-19

**Authors:** Ibrahim Abdullatif Ibrahim

**Affiliations:** ^1^Nursing Department, College of Applied Medical Sciences, Shaqra University, Shaqra 11961, Saudi Arabia; ^2^Nursing Administration Department, Faculty of Nursing, Mansoura University, Mansoura 35516, Egypt

## Abstract

**Aim:**

The objective was to investigate the impact of structural empowerment and demographic factors on the psychological empowerment of nurses.

**Background:**

The empowerment of nurses plays a crucial role in improving patient care and achieving successful healthcare outcomes. When nurses feel empowered in their work environment, they tend to remain dedicated to their jobs and experience higher levels of job satisfaction and engagement.

**Methods:**

This cross-sectional study utilized a convenience sample of 287 nurses recruited from various hospital units, resulting in a response rate of 94.7%. Data were collected through a paper survey consisting of three sections: structural empowerment, psychological empowerment, and demographics.

**Results:**

The first hierarchical regression explains 57.0% variance in nurses' psychological empowerment (*F* = 78.52, *p* < 0.001). Age (*β* = 0.37, *p* < 0.01) and structural empowerment (*β* = 0.69, *p* < 0.001) have a positive influence on nurses' psychological empowerment. Conversely, having a bachelor's degree (*β* = −0.16, *p* < 0.01) had a negative impact psychological empowerment. The second hierarchical regression clarifies the specific aspects of structural empowerment that influences positively on nurses' psychological empowerment: access to opportunities (*β* = 0.13, *p* < 0.01), support (*β* = 0.13, *p* < 0.01), resources (*β* = 0.35, *p* < 0.001), and informal power (*β* = 0.12, *p* < 0.01).

**Conclusion:**

This study emphasizes the importance of considering demographic variables, such as age and education, in conjunction with structural empowerment to effectively enhance nurses' psychological empowerment. *Implications for Nursing Management*. Nursing managers should tailor their empowerment strategies based on the demographic characteristics of their nurses. It is essential to focus on providing nurses with access to opportunities, support, resources, and informal power. These insights provide valuable guidance for nursing managers to enhance nurses' psychological well-being, job satisfaction, and overall quality of life. Ultimately, this contributes to positive outcomes for patients, nursing staff, and healthcare organizations.

## 1. Introduction

The concept of empowerment holds significant importance within contemporary healthcare organizations, serving as a strategy to improve employees' satisfaction with their jobs, motivation, and general well-being [1–3]. The concept of empowerment is multifaceted, encompassing various facets, including structural and psychological empowerment. The notion of structural empowerment refers to the extent to which employees are furnished with resources, knowledge, and support systems that enhance their capacity to effectively fulfill their job duties and exert influence over decision-making processes within the organizational setting [4]. In contrast, psychological empowerment pertains to the subjective perception of individuals in terms of their sense of competence, autonomy, and capacity to exert influence within their work and work environment [5].

Gaining insight into the determinants that impact the nurses' psychological empowerment is of utmost importance, given its significant implications not only on their welfare but also on the quality of patient care delivered [6–9]. Several scholarly investigations were undertaken to evaluate the correlation between structural and psychological empowerment in the healthcare industry. These studies consistently demonstrate the favorable impact of structural empowerment on the psychological empowerment of individuals [2, 10–13]. However, it is imperative to acknowledge that various demographic factors have the potential to impact the psychological empowerment of nurses. Demographic characteristics, such as age, gender, educational level, and years of experience in the nursing profession, have the potential to influence individuals' perceptions of empowerment and their adoption of structural empowerment mechanisms [5, 14, 15].

Despite the growing acknowledgment of the significance of empowerment within the nursing profession, there exists a dearth of scholarly inquiry on the precise ramifications of structural empowerment and demographic factors on Egyptian nurses' psychological empowerment. The existence of this research gap underscores the significance of conducting additional studies to investigate the impact of these factors on the psychological empowerment of hospital nurses in Egypt. By comprehending this correlation, healthcare institutions can enhance their assistance to nursing personnel and foster favorable psychological consequences. Hence, the objective of this research is to investigate the tangible impacts of structural empowerment and demographic factors on Egyptian nurses' psychological empowerment.

## 2. Theoretical Framework

Kanter's theory of empowerment emerges as the most pertinent theory to serve as a guiding framework for this study. According to this theory, four essential conditions facilitate structural empowerment: (1) the provision of opportunities to enhance awareness and skills, (2) access to information regarding the organization's goal or policy, (3) availability of support coupled with gratitude and rewards for proficient tasks, and (4) availability of resources necessary for the effective completion of tasks. This theory posits that structural empowerment materializes when individuals are granted an approach to information, resources, support, and opportunities for learning and development. Consequently, when nurses have access to these invaluable resources and opportunities, they are more likely to experience feelings of competence, autonomy, and control over their work, which, in turn, can foster heightened psychological empowerment [16, 17].

## 3. Literature Review and Hypothesis Development

The role of nurses in the healthcare system is of paramount importance, as they are instrumental in delivering exemplary patient care and facilitating positive healthcare system outcomes. Therefore, the empowerment of clinical nurses, both in terms of structural and psychological aspects, has garnered substantial attention in recent years. This focus stems from the recognition that empowering nurses has the potential to yield far-reaching benefits such as enhanced job satisfaction, improved retention rates, and an overall elevation in the quality of healthcare services [1–3].

### 3.1. Structural Empowerment in Nursing

Structural empowerment is described as the capacity to get things done within the work environment by mobilizing resources, accessing information, and obtaining support to meet organizational goals [16]. Structural empowerment in nursing involves providing nurses with access to information about organization its policies, and procedures, and the work that needs to be carried out, access to the necessary tools, equipment, and other resources to perform job effectively, access to support from supervisor, colleagues, and other members of the organization, and access to opportunities for growth and development to learn new skills to take on new challenge and advance one's career. While informal power is the authority and influence nurses enjoy within the organization, derived from personal ties, competence, reputation, and informal networks. Formal power is bestowed through job designations and hierarchical structures [16].

Structural empowerment holds significant prominence within the nursing profession as it profoundly influences the quality of nurses' work life [6]. Structural empowerment also plays a pivotal role in shaping various facets of nursing practice including the work engagement, psychological empowerment, organizational commitment, and mitigating risk of burnout [4, 5, 11–13, 18–20].

### 3.2. Psychological Empowerment in Nursing

Psychological empowerment is labeled by a motivational condition that comprises four dimensions, namely, meaning, competence, self-determination, and influence [21]. The concept of meaning pertains to the degree to which nurses regard their profession as having significance and being imbued with purpose. Competence refers to the collection of abilities, skills, and aptitudes possessed by nurses, which enable them to carry out their duties and obligations in an effective manner. Self-determination pertains to the nurse's notion of possessing autonomy and liberty in their approach to their professional responsibilities. The notion of impact refers to the perceived degree of influence that the work of nurses exerts on the hospital or department. Spreitzer [21] asserts that management has the potential to significantly contribute to the improvement of the four aspects of psychological empowerment by implementing strategic work design strategies that promote empowerment within the workforce.

Psychological empowerment in the field of nursing is evident through the perception of professionals regarding the worth and recognition attributed to their work, as well as their contributions to the care process. This view gives rise to a feeling of proficiency and autonomy. Hence, it is evident that structural empowerment plays a fundamental role in fostering the psychological empowerment of nurses. Multiple research investigations in nursing have demonstrated the existence of significant associations between structural and psychological empowerment [2, 10–13]. Consequently, the subsequent hypothesis was formulated:

  H1: structural empowerment relates positively to psychological empowerment

The establishment of empowering work conditions is widely acknowledged as a crucial organizational strategy that fosters psychological empowerment, thereby cultivating positive work behaviors and attitudes [22, 23]. Nurses who are affiliated with a specific organization and are bestowed with the necessary information, support, and resources to execute their duties, along with continuous opportunities for growth, are prone to experience an amplified sense of autonomy and job self-efficacy. These elements are pivotal in nurturing psychological empowerment [2]. The ability of an organization to create a framework that allows employees to effectively carry out their tasks by providing them with access to information, support, resources, and opportunities can potentially influence how individuals or groups perceive their psychological empowerment, which includes feelings of meaning, competence, self-determination, and impact [11]. Numerous studies have discovered the interrelations between structural empowerment components and psychological empowerment [5, 7, 12, 24, 25]. Therefore, the following hypotheses were developed:

  H1a: access to opportunities relates positively to psychological empowerment
  H1b: access to information relates positively to psychological empowerment
  H1c: access to support relates positively to psychological empowerment
  H1d: access to resources relates positively to psychological empowerment
  H1e: the formal power of nurses relates positively to psychological empowerment
  H1f: the informal power of nurses relates positively to psychological empowerment

### 3.3. Relationship between Demographic Characteristics and Psychological Empowerment

Demographic factors, such as age, gender, marital status, educational degrees, and experience years, possess the capacity to exert an influence on the perceptions of empowerment among nurses. Previous studies have illuminated that nurses with a greater number of years of experience tend to perceive a heightened sense of empowerment owing to their accumulated knowledge and increased confidence. Similarly, the level of education attained can significantly impact empowerment, as higher levels of education often correlate with greater job autonomy [5, 14, 15]. Consequently, the following hypothesis has been formulated:

  H2: nurses' demographic characteristics relate to psychological empowerment

**Figure 1:**
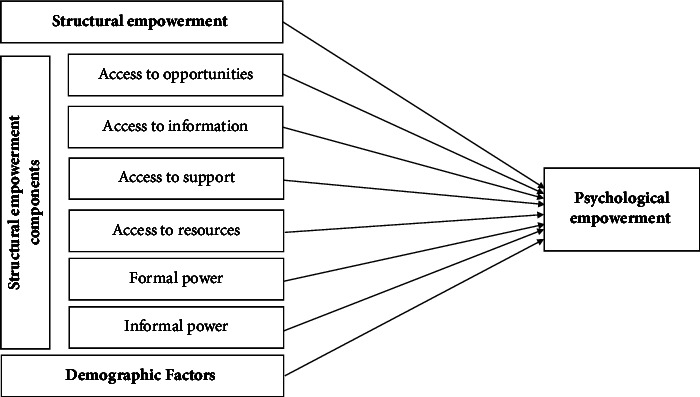
The proposed model of the study.

According to previous literature and theoretical framework, the overall research model of the present study is shown in Figure 1.

## 4. Materials and Methods

### 4.1. Research Design

The study employed a cross-sectional approach using self-reported questionnaires for data collection.

### 4.2. Setting

The study was carried out at a public hospital that is affiliated with the Egyptian Ministry of Health and Population. The hospital provides medical services to a catchment area encompassing an estimated population of 439,000 residents. The hospital provides a comprehensive array of medical treatments encompassing general and specialist surgical procedures, emergency care, cancer treatment, physical therapy and rehabilitation, radiography, and medical laboratory examinations, as well as intensive care.

### 4.3. Participants

This study employed a convenience sampling method whereby participants were selected based on their accessibility and availability within a public hospital affiliated with the Egyptian Ministry of Health and Population. The study included clinical nurses who served in their present nursing role for a minimum of one year, were employed on a full-time basis, and actively engaged in providing direct care to patients. The exclusion criteria encompassed nurses who had been hired for a duration of less than one year, held managerial roles, or served as shift leaders. During work shifts, the participants were approached and invited to partake in the study after a comprehensive explanation of the research objectives. The response rate was an impressive 94.7%, with a total of 287 dedicated male and female clinical nurses willingly participating in the study. The research adhered to the STROBE guidelines.

### 4.4. Instruments of Data Collection

#### 4.4.1. Independent Variable (Structural Empowerment)

The Conditions of Work Effectiveness Questionnaire II (CWEQ-II) was utilized to assess participants' structural empowerment [26]. The instrument utilized in this study comprises six distinct subscales, each measuring a different aspect. These subscales are as follows: access to opportunity (3 items), access to information (3 items), access to support (3 items), access to resources (3 items), formal power (3 items), and informal power (4 items). The participants were tasked with assessing their perceptions of structural empowerment by utilizing a 5-point Likert scale that spanned from 1 (none) to 5 (a lot). The study determined that the internal consistency reliability of the CWEQ-II subscales was deemed satisfactory. The subscales exhibited Cronbach's alpha coefficients ranging from 0.76 to 0.86, while the overall measure of structural empowerment demonstrated Cronbach's alpha coefficient of 0.95.

#### 4.4.2. Dependent Variable (Psychological Empowerment)

The study utilized the 12-item psychological empowerment scale, first established by Spreitzer [21], to evaluate the four distinct components of psychological empowerment. The dimensions encompassed within this framework consist of four key elements: meaning, competence, self-determination, and impact. The psychological empowerment scale comprises three items for each subscale. The respondents provided their responses to the items using a Likert scale with five points, ranging from strongly disagree (1) to strongly agree (5). The subscales of psychological empowerment demonstrated acceptable levels of internal consistency reliability. The presence of Cronbach's alpha coefficients, ranging from 0.72 to 0.83 for the subscales and 0.93 for the overall measure of psychological empowerment, serves as proof for this assertion.

#### 4.4.3. Control Variables (Demographic Variables)

The demographic questionnaire encompasses inquiries concerning age, gender, marital status, experience, education, and the specific unit in which the participants were appointed.

A panel comprising five esteemed experts with diverse backgrounds and experiences was graciously invited to assess the face and content validity of the scales employed in the study. This panel included three professors specializing in nursing management, as well as two experienced clinical nurses. With their meticulous review, minor modifications were thoughtfully incorporated into the scale. For instance, certain questions underwent careful rewording, and explanatory notes were thoughtfully added to specific sections of the questionnaire, thereby enhancing the clarity and comprehensibility of the survey questions.

The pilot study was conducted to refine the scales, thereby yielding more robust measurement instruments that align with the research objectives and target population. Prior research has already established and confirmed the validity and reliability of the CWEQ-II and psychological empowerment scale, demonstrating its effective utilization in nursing populations [2, 9, 11, 27].

### 4.5. Data Collection and Ethical Considerations

The data for this study were acquired through the administration of paper questionnaires to clinical nurses stationed at the research site. The utilization of paper questionnaires was chosen to promote convenience and accessibility for the participants. Several measures were implemented to motivate nurses to diligently complete the survey. Initially, the researcher provided a comprehensive and articulated explanation of the study's aims, emphasizing the potential implications for nursing practice and the enhancement of patient care. Informed consent, signifying the voluntary nature of their involvement, was diligently obtained from each nurse before administrating the questionnaires. Participants were explicitly informed that they had right to withdraw from the study at any time without any consequences or detriment to their employment or participation in future research activities. This process ensured their understanding and willingness to contribute meaningfully to the research endeavor.

Moreover, the participants were assured of the utmost confidentiality regarding their responses. Their invaluable involvement was emphasized, underscoring the significance of their contribution to the study. Importantly, they were informed that their data would be treated with utmost respect for privacy, ensuring that all information provided would be anonymized and kept confidential. The data collection period encompassed the months of February through August 2022, allowing for comprehensive and representative sampling of the clinical nurses' perspectives. Prior to the initiation of data collection, the present study obtained ethical permission from the Ethical Research Committee affiliated with the nursing faculty at Mansoura University, with reference number (Ref.No.P.0449).

### 4.6. Data Analysis

Descriptive statistics were employed to provide a concise summary of the sample characteristics as well as the variables under investigation. Furthermore, independent *t*-tests and ANOVA were utilized to explore potential variations in empowerment levels based on participants' demographic variables. The Pearson product-moment correlation analysis was conducted to assess the relationship between structural and psychological empowerment.

The data analysis employed two instances of regression analysis. The first hierarchical regression analysis aimed to assess the effects of total structural empowerment scores on psychological empowerment (first model). The second hierarchical regression analysis examined the impact of the six constructs of structural empowerment on psychological empowerment (second model). Before conducting these analyses, the demographic variables that demonstrated significant differences in psychological empowerment were considered in the first step of regression analysis. The significance level was set at *p* < 0.05 to ensure rigorous evaluation of the data and precise interpretation of the results. The data analysis was performed using SPSS V23, a widely recognized statistical software package.

## 5. Results

### 5.1. Characteristics of the Studied Clinical Nurses and Differences in Structural and Psychological Empowerment

The majority of clinical nurses were female (92.7%) and married (84.0%). A significant proportion of nurses held technical degrees (56.8%) and worked in inpatient units (68.6%). The participant had a mean age of 31.94 years (SD = 6.54) and a mean of 10.68 years of experience (SD = 7.13). Notably, there were significant differences in both structural and psychological empowerment among different age groups as indicated by the *F*-values of 6.95 (*p* < 0.01) and 11 and 18 (*p* < 0.01) for structural and psychological empowerment, respectively. The age group above 40 years exhibited the highest mean score in structural empowerment (mean = 72.29; SD = 10.69) compared to the age group 31–40 years (mean = 65.95; SD = 14.44) and age group 20–30 years (mean = 61.40; SD = 14.61). The age group above 40 years exhibited the highest mean score in psychological empowerment (mean = 52.38; SD = 5.46) compared to the age group 31–40 years (mean = 48.07; SD = 7.98)and age group 20–30 years (mean = 44.86; SD = 8.02). There were significant differences in psychological empowerment observed between the educational levels of nurses (*F* = 4.50; *p* < 0.05). Nurses with technical education, including those with a diploma or institute nursing degree (referred to as technical nurses), demonstrated significantly higher levels of psychological empowerment (mean = 48.09; SD = 7.44) compared to those with a bachelor's degree (mean = 45.18; SD = 8.80) and postgraduate degree (mean = 46.40; SD = 6.22). Notably, there were significant variations in both structural and psychological empowerment based on diverse levels of experience as indicated by the *F*-values of 6.73 (*p* < 0.01) and 10.86 (*p* < 0.01) for structural and psychological empowerment, respectively. Nurses' with more than 10 years of experience exhibited the highest mean score in structural empowerment (mean = 67.40; SD = 14.39) compared those with 6–10 years of experience (mean = 62.54; SD = 12.73) and 1–5 years of experience (mean = 60.41; SD = 15.42). Nurses' with more than 10 years of experience exhibited the highest mean score in psychological empowerment (mean = 48.91; SD = 7.70) compared those with 6–10 years of experience (mean = 46.37; SD = 7.50) and 1–5 years of experience (mean = 43.81; SD = 8.40) (Table 1).

### 5.2. The Levels of Structural and Psychological Empowerment of Clinical Nurses

The mean score for structural empowerment was 64.24 out of 95 (SD = 14.59), indicating a moderate level of structural empowerment. Among the subscales of structural empowerment, informal power received the highest rating (mean = 2.21, SD = 3.59), while access to information received the lowest rating (mean = 9.44, SD = 3.28). In terms of psychological empowerment, the mean score was 46.85 out of 60 (SD = 8.12), indicating a moderate level of psychological empowerment. Among the dimensions of psychological empowerment, meaning received the highest rating (mean = 12.3, SD = 2.31), while self-determination received the lowest rating (mean = 11.37, SD = 2.50) (Table 2).

### 5.3. Relationship between Structural and Psychological Empowerment of Clinical Nurses

There was a positive and strong relationship between structural and psychological empowerment of clinical nurses (*r* = 0.73, *p* < 0.001), indicating that nurses' perception of structural empowerment increased and their psychological empowerment also increased. In addition, the dimensions of structural empowerment were found to have a positive correlation with psychological empowerment (Table 2).

### 5.4. Influences of Structural Empowerment and Demographic Factors on Nurses' Psychological Empowerment

**Figure 2:**
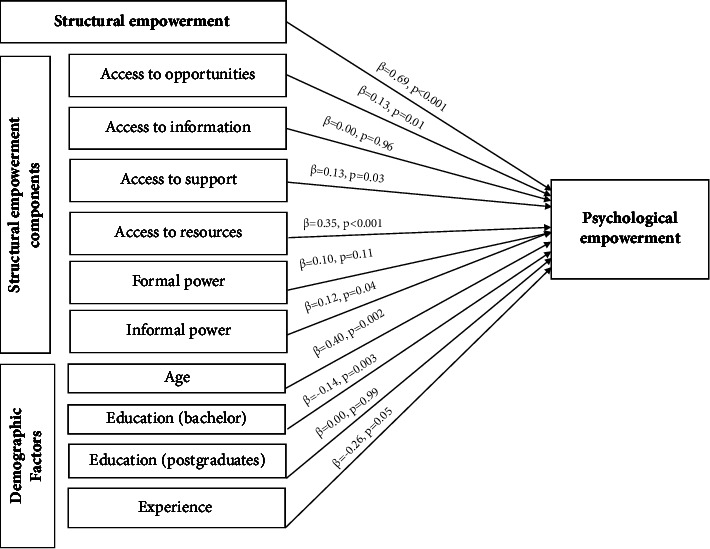
The proposed model of the study with standardized coefficients. Standardized coefficients and actual *p* values of structural empowerment in the first hierarchical regression analysis. Standardized coefficients and actual *p* values of structural empowerment components and demographic factors in the second hierarchical regression analysis.

The first hierarchical regression model accounted for 57.0% of the variance in psychological empowerment (*F* = 78.52, *p* < 0.001). The results revealed that age (*β* = 0.37, *p* < 0.01) and structural empowerment (*β* = 0.69, *p* < 0.001) were positively affecting nurses' psychological empowerment, suggesting that as nurses' age and structural empowerment increase, their psychological empowerment increase. In contrast, the possession of a bachelor's degree (*β* = −0.16, *p* < 0.001) showed a negative association with psychological empowerment, indicating that nurses holding a bachelor's degree exhibited lower psychological empowerment in comparison to their counterparts with technical degrees and postgraduate studies (Table 3 and Figure 2).

The second hierarchical regression model accounted for 60.0% of the variance in psychological empowerment (*F* = 44.38, *p* < 0.001). In terms of demographics, age (*β* = 0.40, *p* < 0.01) and education (bachelor) (*β* = −0.14, *p* < 0.01) were found to be a significant positive factor affecting psychological empowerment. Regarding structural empowerment dimensions, access to opportunities (*β* = 0.13, *p* < 0.01), access to support (*β* = 0.13, *p* < 0.05), access to resources (*β* = 0.35, *p* < 0.001), and informal power (*β* = 0.12, *p* < 0.05) were identified as significant factors affecting nurses' psychological empowerment (Table 4 and Figure 2).

## 6. Discussion

The objective of the study was to examine the influences of structural empowerment and demographic factors on nurses' psychological empowerment, as well as nurses' evaluation of their structural and psychological empowerment.

### 6.1. Nurses' Perceptions of Structural and Psychological Empowerment

The findings revealed a moderate level of both structural and psychological empowerment among nurses, suggesting that they possess partial developmental opportunities, support systems, and resources that contribute to their competence and job satisfaction. These outcomes align with previous research conducted in diverse countries. For instance, Tan and Conde [15] conducted a study on Filipino nurses and reported moderate levels of empowerment in both their qualities and performances. Moura et al. [28] found a moderate level of empowerment among nurses working in a teaching hospital located in southern Brazil. In a cross-sectional study by Yu et al. [29], Chinese nurses in tertiary hospitals were found to have moderate levels of psychological empowerment. Khrais and Nashwan [27] investigated emergency nurses in three large hospitals in Jordan and observed a moderate level of perceived structural empowerment and a high-moderate level of perceived psychological empowerment. Similarly, a study by Walden [30] revealed moderate levels of structural empowerment, psychological empowerment, clinical nurse educator leadership, and work engagement among new graduate nurses in acute care settings. Saleh et al. [3] explored the experiences of nurses in two hospitals in Jordan and found a moderate level of structural empowerment reported by the participants. Furthermore, a study by Di Napoli et al. [31] showcased high levels of both structural and psychological empowerment among nurses attending the National Association of Orthopedic Nursing annual conference. In contrast, Saleh et al. [3] reported a low degree of psychological empowerment among nurses in two hospitals in Jordan.

Within the realm of structural empowerment, the study identified that informal power received the highest rating among various constructs. This finding suggests that nurses place immense value on personal relationships, considering them to be more influential than other aspects of structural empowerment. Conversely, access to information was rated the lowest, indicating that nurses perceive limited availability of pertinent information. Prior research conducted in Iran, Saudi Arabia, and Bangladesh has yielded equivalent results regarding nurses' perception of access to opportunities and support being the highest-rated components, while formal power tends to receive the lowest ratings [28, 32]. In a study by Orgambídez-Ramos et al. [18], access to opportunities and informal power were also ranked highest, while access to resources received the lowest rating among the components of structural empowerment.

Regarding the components of psychological empowerment, the nurses in this study ranked meaning as the most significant factor. This finding suggests that nurses derive a sense of purpose and fulfillment from their work, recognizing the value and significance of their roles. On the other hand, the impact was deemed the least significant, indicating that nurses perceive themselves as having less influence over their work environment and decision-making processes. These findings align with previous studies conducted by Di Napoli et al. [31] and Kebriaei et al. [33], which also highlighted the importance of meaning in nurses' psychological empowerment. However, it is worth noting that Saleh et al. [3] found that competency ranked the highest among the psychological empowerment factors, followed by meaning, self-determination, and impact. In addition, Moosavi et al. [34] reported that competence was perceived as the most significant factor, while self-determination and trust were rated the lowest.

### 6.2. Influences of Structural Empowerment and Demographic Factors on Nurses' Psychological Empowerment

The study findings revealed a positive influence of structural empowerment and older age on nurses' psychological empowerment, supporting hypothesis 1 (H1) and partially hypothesis 2 (H2). Structural empowerment particularly in terms of access to opportunities, support, resources, and informal power had positive influences on nurses' psychological empowerment, thereby providing support for hypotheses 1 (H1), H1a, H1c, H1d, and H1f. These findings can be justified through various mechanisms. First, access to opportunities allows nurses to participate in professional development programs and acquire new skills contributing to their sense of competence and the confidence in their rules. Second, support they received from colleagues and supervisors fosters a supportive work environment, where their contributions are valued leading to increased empowerment. Certainly, having access to essential resources such as information and the tools enhances necessary efficiency and effectiveness, reducing potential sources of frustration and disempowerment. Lastly, informal power enables nurses to have a voice in a decision-making process, giving him a sense of influence and autonomy over their work. These combined factors illustrate multifaceted nature of structure empowerment in positively impacting nursing psychological empowerment which in turn can enhance their job satisfaction and well-being. However, it was observed that nurses with a bachelor's degree showed a negative association with psychological empowerment, indicating that they may experience lower levels of empowerment compared to those with different educational backgrounds (partially support H2). This negative association between nurses holding a bachelor's degree and psychological empowerment may be due to transition shock and discrepancies in role expectations. Nurses with higher educational qualifications may have higher expectations of their roles and responsibilities, which can lead to feelings with disempowerment if these expectations are not met in practice. The study by Karkkola et al. [35] revealed that there exists a positive relationship between role clarity and subjective vitality in the workplace, which can be attributed to increased levels of autonomy and competence. Conversely, a negative association was observed between role conflict and subjective vitality at work, which can be attributed to decreased levels of autonomy and relatedness.

These findings align with previous research conducted by Aggarwal et al. [36], which demonstrated a positive correlation between structural empowerment and its constructs with psychological empowerment, except for access to resources, which showed a negative correlation. Similarly, Di Napoli et al. [31] reported a positive correlation between structural and psychological empowerment. Stewart et al. [19] also found consistent results, confirming a positive correlation between access to opportunities and support and nurses' psychological empowerment. Furthermore, meta-analysis research conducted by Zhang et al. [5] supported a positive correlation between structural and psychological empowerment in nurses. Orlowska and Laguna [2] conducted a research study that indicated a positive relationship between structural empowerment at the hospital department level and nurses' sense of competence and autonomy, which is referred to as psychological empowerment. Empirical studies examining the mediating role of psychological empowerment in the presence of structural empowerment as an independent variable also supported the positive relationship between structural and psychological empowerment [1, 6, 10, 13]. In addition, Zeb et al. [37] identified three main themes that influence nurse educators' psychological empowerment: poor organizational structure, dynamics of educators-academic administrators' relations, and educational tools and physical environment.

The relationship between structural empowerment and psychological empowerment yields numerous benefits for individuals and organizations alike. When employees feel empowered, they are more likely to experience increased job satisfaction, motivation, and engagement [3, 22]. They also tend to exhibit greater creativity, productivity, and innovation. By investing in structural empowerment initiatives, organizations can create a positive work culture that fosters psychological well-being and drives overall success [4, 8, 14, 27]. In this line, the study by He et al. [38] reported that nurses' work engagement serves as an important mechanism through which their psychological capital influences the satisfaction of elderly cancer patients. The relationship between nurses' psychological capital and work engagement is also positively moderated by job resources, indicating that the presence of these employment resources amplifies the influence of nurses' psychological capital on their level of work engagement. This finding suggests that workplace resources serve as enhancers, intensifying the relationship between nurses' positive psychological perspective and their level of work engagement [38]. Recent mediation studies revealed that structural empowerment had a positive effect on psychological empowerment that ultimately led to positive consequences. In other words, psychological empowerment is an underlying mechanism that explains why structural empowerment is positively related to positive outcomes. For instance, the study by Yu et al. [29] found that psychological empowerment plays a mediating role in the relationship between nurses' perceptions of decent work and work immersion. The study by Şenol Çelik et al. [9] found that both structural and psychological empowerment can reduce burnout among nurses and improve patient care quality. Psychological empowerment played a partially mediating role in the relationship between transformational leadership and burnout [39].

The present study is in the same line with Ouyang et al. [40], who reported that psychological empowerment was significantly different concerning age and length of service among nurses. Also, Browning [41] showed a positive connection between psychological empowerment and age, experience, collaboration in end-of-life care conferences, and education. Empirical research studies confirmed that there were no significant relationships found between age, gender, marital status, educational level, hospital-employed (community, academic, or other), type of employment (permanent or temporary), years in the profession, years worked in present facility, or type of unit worked on (medical, surgical, intensive care, obstetrics, pediatrics, operating room, postanesthetic care, psychiatry, emergency, ambulatory care, or other) and structural and psychological empowerment [11, 42, 43]. The Filipino study by Tan and Conde [15] revealed that a nurse's level of empowerment increases as the nurse grows older who becomes tenured at work and embodies higher empowerment qualities and performances.

### 6.3. Limitations and Future Research

While this study provides valuable insights, it is essential to acknowledge its limitations. The utilization of a cross-sectional design imposes limitations on our capacity to demonstrate causal linkages over an extended period. The utilization of a convenience sample derived exclusively from hospital units may impose constraints on the extent to which the findings can be extrapolated to a more comprehensive healthcare setting. Moreover, the utilization of self-reported data entails the possibility of encountering response and social desirability biases. Therefore, longitudinal studies can provide insights into nurse empowerment over time, identifying key points in their careers for effective interventions. Comparative research across different healthcare settings can uncover variations in the relationships between empowerment, demographics, and psychological empowerment, informing targeted interventions. Qualitative studies can explore the experiences of empowered nurses, offering nuanced insights for organizational improvements. Intervention studies can evaluate the impact of empowerment initiatives on nurses' psychological empowerment and patient care outcomes, providing evidence-based interventions. Multinational studies can explore how cultural factors influence empowerment and demographics, contributing to a comprehensive understanding. Lastly, researching the direct impact of nurse empowerment on healthcare outcomes can provide compelling evidence of the connection between empowerment and enhanced delivery of healthcare services.

### 6.4. Implications of the Study

The insights from this study offer valuable information for policymakers and clinical managers. They shed light on the significance of addressing clinical nurses' needs for access to opportunities, resources, support, and informal power, as satisfying these needs will enhance nurses' psychological empowerment and job performance. This understanding can inform policy decisions and managerial strategies aimed at improving job satisfaction, motivation, and nurses' sense of meaning and competence, all of which contribute to better patient outcomes.

Furthermore, the study underscores the importance of considering investments in structural initiatives such as the development of clear career paths and professional development opportunities as means to support nursing staff effectively. Also, the negative impact of holding bachelor's degrees on nurses' psychological empowerment can be addressed. These findings suggest that healthcare organizations may need to align job roles and responsibilities with qualifications of their nursing staff. Healthcare managers should implement mentorship program and leadership development initiatives that may help bridge this gap and foster psychological empowerment, regardless of their educational qualifications. Nursing programs should incorporate elements that enhance psychological empowerment such as resilience, coping strategies, problem solving, self-reflection, and self-efficacy.

## 7. Conclusions

In this study, we examined the influences of structural empowerment and demographic factors on nurses' psychological empowerment. Our findings shed light on the complex interplay of these variables within the context of healthcare settings. The key conclusions drawn from our research are as follows: (1) This study found a significant positive association between structural empowerment and psychological empowerment among clinical nurses. These findings suggest that nurses who perceive themselves as structurally empowered are more likely to experience higher levels of psychological empowerment. (2) The current study highlighted the impact of demographic factors on psychological empowerment. Age emerged as a positive predictor, indicating that older nurses tend to report higher psychological empowerment. Conversely, having a bachelor's degree was associated with lower psychological empowerment. These findings underscore the importance of considering demographic variables in understanding nurses' empowerment. (3) This study also revealed that access to opportunities, access to support, access to resources, and informal power dimensions of structure empowerment were identified as significant predictors of psychological empowerment. These dimensions represent critical aspects of the work environment that influence nurses' sense of empowerment. (4) The study found that clinical nurses reported moderate levels of both structural and psychological empowerment. (5) Access to resources received the highest rating among structural empowerment dimensions, while meaning was the highest-rated dimension of psychological empowerment. These insights can inform interventions aimed at enhancing empowerment levels.

## Figures and Tables

**Table 1:** Demographic characteristics of the studied clinical nurses and differences in structural and psychological empowerment.

Characteristics	*N* (%)	Structural empowerment	*t* or *f*	Psychological empowerment	*t* or *f*
		Mean ± SD		Mean ± SD	
Age years					
20–30	137 (47.7)	61.40 ± 14.61	6.95^*∗∗*^	44.86 ± 8.02	11.18^*∗∗*^
31–40	129 (44.9)	65.95 ± 14.44		48.07 ± 7.98	
>40	21 (7.3)	72.29 ± 10.69		52.38 ± 5.46	
Mean ± SD	31.92 ± 6.54				
Gender					
Male	21 (7.3)	67.95 ± 11.51	1.21	49.43 ± 6.00	1.51
Female	266 (92.7)	63.95 ± 14.78		46.65 ± 8.24	
Marital status					
Unmarried	46 (16.0)	64.78 ± 13.11	0.27	45.98 ± 7.64	
Married	241 (84.0)	64.14 ± 14.88		47.02 ± 8.21	0.80
Levels of education					
Technical degree	163 (56.8)	65.65 ± 13.16		48.09 ± 7.44	
Bachelor's degree	119 (41.5)	62.54 ± 16.00	1.91	45.18 ± 8.80	4.50^*∗*^
Postgraduate degree	5 (1.7)	59.00 ± 21.19		46.40 ± 6.22	
Experience years					
1–5	81 (28.2)	60.41 ± 15.42		43.81 ± 8.40	
6–10	70 (24.4)	62.54 ± 12.73	6.73^*∗∗*^	46.37 ± 7.50	10.86^*∗∗*^
>10	136 (47.4)	67.40 ± 14.39		48.91 ± 7.70	
Mean ± SD	10.68 ± 7.13				
Unit					
Inpatient units	197 (68.6)	64.63 ± 14.65	0.66	47.33 ± 8.19	1.47
ICUs	90 (31.4)	63.40 ± 14.50		45.81 ± 7.92	

^*∗*^ *p* < 0.05, ^*∗∗*^*p* < 0.01. ICUs: intensive care units.

**Table 2:** Descriptive statistics and correlations between structural and psychological empowerment among the studied clinical nurses.

	Min-max	Mean ± SD	1	2	3	4	5	6	7	8	9	10	11
(1) Structure empowerment	24–95	64.24 ± 14.59											
(2) Access to opportunities	3–15	11.01 ± 2.67	0.78^*∗∗∗*^										
(3) Access to information	3–15	9.44 ± 3.28	0.79^*∗∗∗*^	0.51^*∗∗∗*^									
(4) Access to support	3–15	10.56 ± 2.72	0.83^*∗∗∗*^	0.66^*∗∗∗*^	0.59^*∗∗∗*^								
(5) Access to resources	3–15	11.20 ± 2.93	0.80^*∗∗∗*^	0.59^*∗∗∗*^	0.51^*∗∗∗*^	0.60^*∗∗∗*^							
(6) Formal power	3–15	9.83 ± 2.62	0.84^*∗∗∗*^	0.58^*∗∗∗*^	0.64^*∗∗∗*^	0.61^*∗∗∗*^	0.61^*∗∗∗*^						
(7) Informal power	4–20	12.21 ± 3.59	0.85^*∗∗∗*^	0.56^*∗∗∗*^	0.56^*∗∗∗*^	0.66^*∗∗∗*^	0.61^*∗∗∗*^	0.71^*∗∗∗*^					
(8) Psychological empowerment	16–60	46.85 ± 8.12	0.73^*∗∗∗*^	0.59^*∗∗∗*^	0.50^*∗∗∗*^	0.62^*∗∗∗*^	0.69^*∗∗∗*^	0.60^*∗∗∗*^	0.61^*∗∗∗*^				
(9) Meaning	5–15	12.31 ± 2.31	0.61^*∗∗∗*^	0.53^*∗∗∗*^	0.35^*∗∗∗*^	0.54^*∗∗∗*^	0.62^*∗∗∗*^	0.47^*∗∗∗*^	0.49^*∗∗∗*^	0.85^*∗∗∗*^			
(10) Competence	5–15	12.00 ± 2.16	0.52^*∗∗∗*^	0.49^*∗∗∗*^	0.32^*∗∗∗*^	0.46^*∗∗∗*^	0.53^*∗∗∗*^	0.37^*∗∗∗*^	0.43^*∗∗∗*^	0.84^*∗∗∗*^	0.68^*∗∗∗*^		
(11) Self-determination	3–15	11.37 ± 2.50	0.68^*∗∗∗*^	0.50^*∗∗∗*^	0.51^*∗∗∗*^	0.54^*∗∗∗*^	0.61^*∗∗∗*^	0.58^*∗∗∗*^	0.59^*∗∗∗*^	0.87^*∗∗∗*^	0.61^*∗∗∗*^	0.65^*∗∗∗*^	
(12) Impact	3–15	11.17 ± 2.52	0.69^*∗∗∗*^	0.51^*∗∗∗*^	0.51^*∗∗∗*^	0.58^*∗∗∗*^	0.60^*∗∗∗*^	0.61^*∗∗∗*^	0.56^*∗∗∗*^	0.85^*∗∗∗*^	0.62^*∗∗∗*^	0.59^*∗∗∗*^	0.69^*∗∗∗*^

^*∗∗∗*^ *p* < 0.001.

**Table 3:** Influence of structural empowerment on psychological empowerment of the studied clinical nurses.

	Unstandardized coefficients		Standardized coefficients	*t*
	*B*	Std. error	*β*	
Constant	11.45	3.70		3.10^*∗∗*^
Age	0.46	0.17	0.37	2.76^*∗∗*^
Education^a^				
Education (bachelor)	−2.61	0.81	−0.16	−3.21^*∗∗*^
Education (postgraduates)	−0.06	2.44	0.00	−0.03
Experience	−0.26	0.15	−0.23	−1.69
Structure empowerment	0.38	0.02	0.69	17.17^*∗∗∗*^
*R*, *R*^2^, adj. *R*^2^, *F*	0.76, 0.58, 0.57, 78.52^*∗∗∗*^			

^*∗∗*^ *p* < 0.01, ^*∗∗∗*^*p* < 0.001. ^a^: dummy reference group, technical education degree.

**Table 4:** Influence of structural empowerment factors on psychological empowerment of the studied clinical nurses.

	Unstandardized coefficients		Standardized coefficients	*t*
	*B*	Std. error	*β*	
Constant	8.96	3.68		2.43^*∗*^
Age	0.50	0.16	0.40	3.08^*∗∗*^
Education^a^				
Education (bachelor)	−2.37	0.79	−0.14	−3.01^*∗∗*^
Education (postgraduates)	0.04	2.37	0.00	0.02
Experience	−0.30	0.15	−0.26	−1.98
Access to opportunities	0.41	0.16	0.13	2.50^*∗*^
Access to information	0.01	0.13	0.00	0.05
Access to support	0.40	0.18	0.13	2.21^*∗*^
Access to resources	0.98	0.15	0.35	6.61^*∗∗∗*^
Formal power	0.31	0.19	0.10	1.63
Informal power	0.28	0.13	0.12	2.08^*∗*^
*R*, *R*^2^, adj. *R*^2^, *F*	0.79, 0.62, 0.60, 44.38^*∗∗∗*^			

^*∗*^ *p* < 0.05, ^*∗∗*^*p* < 0.01, ^*∗∗∗*^*p* < 0.001. ^a^: dummy reference group, technical education degree.

## Data Availability

The data used to support the study are available from the corresponding author upon request.
